# Nephrotic Syndrome Complicated by Pulmonary Embolism and Inferior Vena Cava Thrombosis: A Case Report

**DOI:** 10.7759/cureus.71698

**Published:** 2024-10-17

**Authors:** Yousef M Hamed, Ahmed M Hamed, Maha A Younes, Mahmoud I Ahmed, Mostafa Ebraheem Morra

**Affiliations:** 1 College of Medicine, Alfaisal University, Riyadh, SAU; 2 Faculty of Radiology, Aberdeen Royal Infirmary, Aberdeen, GBR; 3 Internal Medicine, Warrington General Hospital, Warrington and Halton Hospitals NHS Foundation Trust, Warrington, GBR; 4 Cardiology, East Sussex Healthcare NHS Trust, East Sussex, GBR; 5 Faculty of Medicine, Pilgrim Hospital, United Lincolnshire Hospitals NHS Trust, Boston, GBR

**Keywords:** case report, deep vein thrombosis (dvt), inferior vena cava thrombus, nephrotic syndrome, pulmonary embolism

## Abstract

This article presents the case of a 52-year-old male who arrived at the emergency department with escalating chest pain and shortness of breath. An urgent CT scan revealed a right pulmonary embolism and inferior vena cava thrombus, prompting immediate anticoagulant therapy. Further diagnostic evaluations confirmed nephrotic syndrome (NS). The patient was discharged on apixaban, ramipril, amlodipine, and atorvastatin. A subsequent renal biopsy showed features consistent with minimal change disease, which resolved spontaneously without the need for steroid treatment. This case highlights the potentially life-threatening presentation of multiple venous thromboses as an initial manifestation of NS.

## Introduction

Nephrotic syndrome (NS) is defined as proteinuria (≥3.5 g/24 hours), hypoalbuminemia (<3.0 g/dL), peripheral oedema, hyperlipidaemia, and high thrombosis risk [1]. NS is classified into primary, which includes focal segmental glomerulosclerosis, membranous nephropathy, and minimal change disease, or secondary, which could be drug-related such as penicillamine and pamidronate or systemic disease-related such as malignancy, diabetes mellitus, and systemic lupus erythematosus [2]. The estimated incidence of NS is three cases per 100,000 in adults and two to seven cases per 10,000 in children [2]. NS-related complications include infections (e.g., sepsis), thromboembolism, cardiovascular problems (e.g., hyperlipidaemia), renal failure, and anaemia [3]. Thromboembolism including pulmonary embolism (PE), renal vein thrombosis, and deep vein thrombosis (DVT) tend to develop in NS and have been considered life-threatening complications. Augmented platelet aggregation and loss of anticoagulants such as antithrombin III are thought to enhance the propensity to venous thrombosis in NS [4,5]. Here, we present the case of a middle-aged healthy man with PE and inferior vena cava (IVC) thrombus as a presenting feature of NS.

## Case presentation

A 52-year-old healthy male was admitted to the emergency department with complaints of shortness of breath and sharp right-sided chest pain radiating to his back. The symptoms had been worsening over the past few days before admission and were complicated by coughing streaks of blood. The patient had never been to a hospital with similar complaints, and his previous medical history was only positive for type 2 diabetes mellitus managed by diet modification.

On closer assessment, the pain was noted to be pleuritic, and bilateral mild pitting oedema was noted in both lower limbs. The rest of the physical examination was unremarkable. The National Early Warning Score was 0, pulse rate was 78 beats/minute, respiratory rate was 20 breaths/minute, blood pressure was 150/76 mmHg, and oxygen saturation was 96% on room air. Initial laboratory investigations revealed urea of 8.1 mmol/L and creatinine levels of 159 µmol/L with a glomerular filtration rate (GFR) of 42 mL/minute. It also showed a C-reactive protein level of 163 mg/L, D-dimer of 805 ng/mL, albumin of less than 10 g/L, and total protein of 57 g/L. Other laboratory tests such as complete blood count, liver function tests, serum electrolytes, and lipid profile were within the normal range (Table 1).

**Table 1:** Complete blood count, liver function, serum electrolytes, lipid profile, and thrombophilia screening results.

Test	Result	Reference range
Complete blood count		
Haemoglobin	14.5 g/dL	13.0–17.0 g/dL
White blood cell count	6.8 × 10^9^/L	4.0–11.0 × 10^9^/L
Platelet count	250 × 10^9^/L	150–450 × 10^9^/L
Liver function tests		
Alanine aminotransferase	30 U/L	7–56 U/L
Aspartate aminotransferase	25 U/L	10–40 U/L
Alkaline phosphatase	90 U/L	44–147 U/L
Total bilirubin	0.8 mg/dL	0.1–1.2 mg/dL
Serum electrolytes		
Sodium	140 mmol/L	135–145 mmol/L
Potassium	4.2 mmol/L	3.5–5.1 mmol/L
Chloride	102 mmol/L	98–107 mmol/L
Bicarbonate	24 mmol/L	22–28 mmol/L
Lipid profile		
Total cholesterol	180 mg/dL	<200 mg/dL
Low-density lipoprotein	90 mg/dL	<100 mg/dL
High-density lipoprotein	55 mg/dL	>40 mg/dL
Triglycerides	120 mg/dL	<150 mg/dL
Thrombophilia screening		
Antithrombin level	70 IU/dL	80–120 IU/dL
Protein C	Normal	Normal
Protein S	Normal	Normal
Factor V Leiden	Negative	Negative
Anti-β-2-glycoprotein-1 antibodies	Negative	Negative
Anticardiolipin antibodies	Negative	Negative
Phospholipase A2 receptor antibody	Negative	Negative
Antinuclear antibody	Negative	Negative
Antineutrophil cytoplasmic antibody	Negative	Negative
Plasma Homocysteine	Normal	Normal

**Figure 1:**
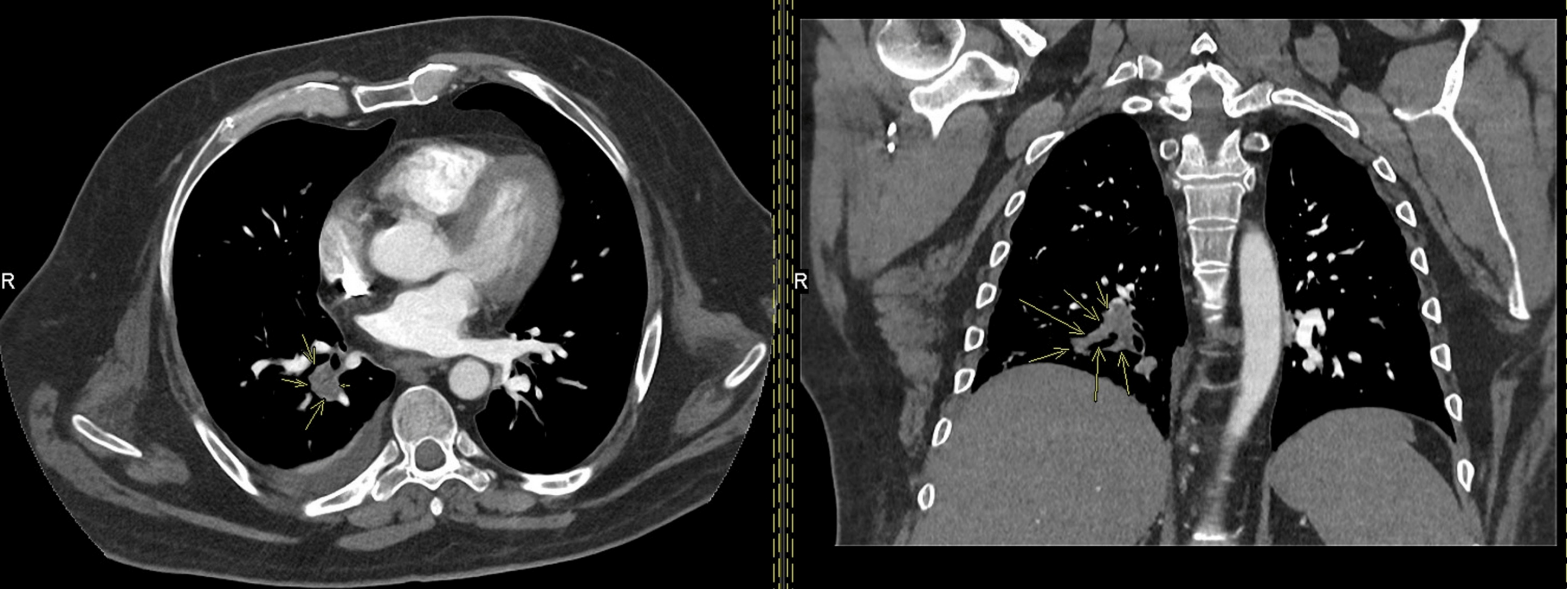
CT pulmonary angiography revealing filling defects (yellow arrows) in multiple segmental and subsegmental branches on the right side.

**Figure 2:**
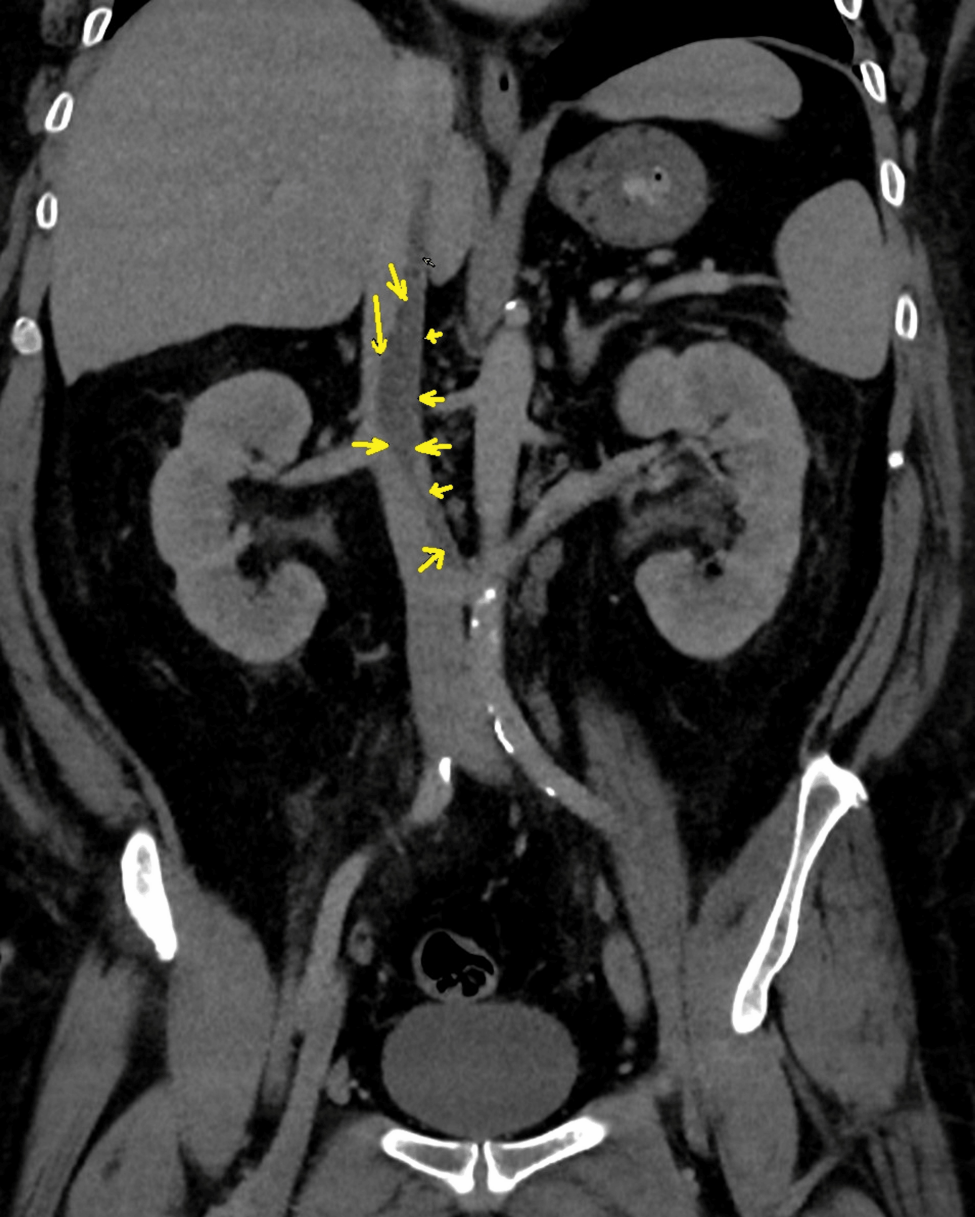
CT showing a long irregular eccentric filling defect (yellow arrow) in the inferior vena cava.

The patient received initial doses of intravenous broad-spectrum antibiotics and subcutaneous low-molecular-weight heparin for a probable chest infection and PE, respectively. Urgent CT pulmonary angiogram (CTPA) revealed filling defects in multiple segmental and subsegmental branches on the right side with mild pleural effusion and basal atelectasis on the same side (Figure 1). The scan also showed a long irregular eccentric filling defect in the IVC, representing an IVC thrombus. A CT scan of the abdomen/pelvis with contrast was unremarkable (Figure 2).

Further laboratory investigations showed that urine protein was 6,151 mg/L, urine albumin >400 mg/L, 24-hour urine albumin >590 mg/24 hour, and 24-hour urine volume was 1.47L. The vital signs showed blood pressure persistently above 160/85 mmHg during admission days. The kidney function remained stable with a GFR of around 40 mL/minute. Thrombophilia screening revealed an antithrombin level of 70 IU/dL. Protein C, protein S, factor V Leiden, anti-β-2-glycoprotein-1 antibodies, anticardiolipin antibodies, phospholipase A2 receptor antibodies, antinuclear antibodies, antineutrophil cytoplasmic antibodies, and plasma homocysteine were all negative or within the normal range (Table 1). Screening for human immunodeficiency virus, hepatitis B virus, hepatitis C virus, varicella-zoster virus, QuantiFERON-TB Gold, and Rubella was also within the normal range or negative (Table 2).

**Table 2:** Infectious disease screening results and immunity status.

Test	Result	Reference range
Human immunodeficiency virus (antigen/antibody)	Negative	Negative
Hepatitis B (HBsAg)	Negative	Negative
Hepatitis C (anti-HCV)	Negative	Negative
Varicella-Zoster virus IgG	180 IU/L	>150 IU/L (immunity)
QuantiFERON-TB Gold	0.10 IU/mL	<0.35 IU/mL (negative)
Rubella IgG	25 IU/mL	>10 IU/mL (immunity)

The renal team was consulted, who requested the workup mentioned above and arranged an outpatient follow-up. The patient was diagnosed with hypertension and NS of unknown cause leading to thromboembolism. After two weeks of admission, he was deemed medically fit to be discharged on the following medications: apixaban 5 mg BD, ramipril 2.5 mg OD, amlodipine 10 mg OD, and atorvastatin 20 mg OD.

Follow-up three months after discharge by the renal team revealed persistent high protein in the urine, low serum albumin, and chronic kidney disease stage 3 G3bA3 (Table 3). Renal biopsy at six months of discharge, after stopping apixaban, showed features suggestive of minimal change disease. Thus, the patient was not commenced on steroids given the impression of spontaneous remission of the disease, with a plan to follow up and repeat the urine protein test in four weeks.

**Table 3:** Urine protein, serum albumin, and chronic kidney disease stage results at the three-month follow-up.

Test	Result	Reference Range
Urine protein		
24-hour urine protein	4.5 g/day	<150 mg/day
Protein-creatinine ratio	500 mg/mmol	<15 mg/mmol
Serum albumin	2.8 g/dL	3.5–5.0 g/dL
Chronic kidney disease (CKD)		
Estimated glomerular filtration rate (eGFR)	35 mL/minute/1.73 m²	≥90 mL/minute/1.73 m²
CKD stage	3 G3bA3	G3b: eGFR 30–44 mL/minute/1.73 m² (A3: high albuminuria)

## Discussion

NS carries a high risk of thromboembolism such as PE, DVT, and IVC thrombus which are deemed serious complications [4,5]. The triggers of hypercoagulability in NS include antithrombotic factor imbalance, depletion of intravascular volume, immobilization, and protein C and protein S deficiencies [6]. A recent meta-analysis of 11 studies by Leslom et al. showed that the overall prevalence of PE in patients with NS is 7.93%; however, there was significant heterogeneity between the included studies. The authors also highlighted the value of early management in curbing the mortality burden [7]. In a case report by Jeele et al., a 19-year-old female presented with chest pain and difficulty breathing, and her CT scan showed PE and IVC thrombus due to NS, allowing immediate anticoagulation [8]. The patient was hospitalized for two weeks, her symptoms were relieved, her laboratory values nearly returned to normal, and she was discharged on steroids, statins, warfarin, and ramipril. A follow-up after six months showed no further complaints and no thrombus in the repeat CT scan [8]. In another report by Mohamed et al., a 60-year-old female, with a history of diabetes mellitus, presented with breathlessness and lower limb oedema for two weeks [9]. The CT angiography showed a thrombus at the right main pulmonary artery along with IVC thrombosis extending in both renal veins, the right common and left common iliac vein, as a complication of NS. The patient was commenced on heparin and warfarin and methylprednisone, and furosemide was also initiated for her moderate ascites. The patient later became clinically stable and was discharged with a follow-up plan [9].

Despite the lack of conclusive evidence, corticosteroids have been often used in the treatment of NS [10]. Immunosuppressive treatments may be considered under the advice of a nephrologist for patients who are steroid-resistant or do not improve [9,10]. In our study, the patient was not commenced on steroids and was planned for a renal biopsy first after stopping the anticoagulant. While his minimal change disease showed spontaneous remission, steroids were administered. Earlier identification of the disease through renal biopsy and timely initiation of steroids might have resulted in a more rapid improvement. It is noteworthy to mention that preventing thrombosis with oral anticoagulants in NS patients necessitates a comprehensive case-by-case evaluation for the risks of further thromboembolic events and drug-induced bleeding [11].

## Conclusions

This report illustrates the subtle presentation that multiple venous thromboses might have as a first presenting feature in patients with NS which may be life-threatening. All physicians should have clinical suspicion for such cases to provide earlier diagnosis and effective treatment.
